# Clinicoradiological evaluation of oxidative stress activity in acute cerebral infarction in the first 24 h and the qualitative importance of dysfunctional HDL in stroke

**DOI:** 10.55730/1300-0144.5539

**Published:** 2022-08-30

**Authors:** Esra DEMİR ÜNAL, Gönül VURAL, Funda EREN, Salim NEŞELİOĞLU, Özcan EREL

**Affiliations:** 1 Department of Neurology, Nevşehir State Hospital, Nevşehir, Turkey; 2 Department of Neurology, Faculty of Medicine, Ankara Yıldırım Beyazıt University, Ankara, Turkey; 3 Department of Medical Biochemistry, Ankara City Hospital, Ankara, Turkey; 4 Department of Medical Biochemistry, Faculty of Medicine, Ankara Yıldırım Beyazıt University, Ankara, Turkey

**Keywords:** Dysfunctional HDL, infarct volume, lipid hydroperoxide, myeloperoxidase, oxidative stress

## Abstract

**Background/aim:**

Acute cerebral infarction (ACI) occurs as a result of instant disruption of vascular flow that causes disbalance between oxidative/antioxidative activity. We examined the relationship of serum neuro-oxidative stress parameters with stroke severity and infarct volume in ACI and emphasized the qualitative importance of high-density lipoprotein (HDL) on its relationship with myeloperoxidase (MPO) and paraoxonase-1 (PON1) in the acute period of stroke.

**Materials and methods:**

One hundred ACI patients applied within the first 24 h and 50 healthy volunteers were included. The patient group was evaluated with demographic data (including arrival serum biochemical assessment), clinical disability scores, infarct volume, serum oxidative/antioxidative parameters (lipid hydroperoxide (LOOH), MPO, PON1, MPO/PON ratio). The relevant serum parameters were compared with the control group. Dysfunctional HDL measurement was based on detecting dysfunctionality as a result of a high positive correlation between the dysfunctional feature of HDL and the MPO/PON ratio. The correlation of serum parameters, clinical disability score, and infarct volume were evaluated, and independent analyses of variability with comorbidities were performed.

**Results:**

A negative correlation between PON1 and arrival NIH score/scale (NIHSS), LOOH and discharge modified rankin scale (mRS), triglyceride level, and infarct volume; a positive correlation between MPO\PON ratio and infarct volume was determined. Logistic regression analyses showed that hypertension, diabetes, and high HbA1C may be predictors of stroke severity, and diabetes mellitus, high HbA1C, infarct volume, and high NIHSS score may be predictors of early disability (p < 0.005). The ROC curve analysis revealed that determining the cut-off value for LOOH is of importance in determining early disability scores (7.2 and 6.2, respectively).

**Conclusion:**

The balance between oxidative and antioxidative stress parameters and their quantitative/qualitative changes is of importance, especially in the acute period of ACI. Dysfunctional HDL’s evolution and its relationship with other oxidants are significant not only in the cardiovascular aspect but also in the clinicoradiological aspect.

## 1. Introduction

Stroke, according to the definition of the World Health Organization, is a clinical syndrome characterized by a rapid settlement of signs and symptoms of focal loss of cerebral function for no apparent reason other than vascular causes. Symptoms last longer than 24 h and may result in death [1]. Stroke can be classified into 2 categories, hemorrhagic and ischemic, the latter being the most prevalent form accounting for up to 87% of all cases [2]. A significant proportion of ischemic stroke is caused by atherothrombosis, defined as atherosclerotic plaque disruption with superimposed thrombosis [3].

Unlike other organs, the brain is especially vulnerable to ROS and RNS increases due to low neuron antioxidant enzyme activity and high concentrations of peroxidizable lipids, high O_2_ consumption, and high levels of iron, all acting as prooxidants under pathological conditions [4]. In agreement with this view, ROS production has been reported as an important mechanism of brain injury after exposure to ischemia and reperfusion [5].

High-density lipoproteins (HDLs) are complex particles containing multiple proteins that have been reported to be modified under pathological conditions [6–9]. Although it has been shown in several studies that the functionality of HDL rather than HDL level is effective on cardiovascular disease risk in cardiovascular diseases, there are not enough studies yet in this area in stroke patients.

In this study, we aimed to reveal whether the severity of the stroke, the extent of ischemia, and atherosclerosis is related to HDL level or its functional capacity, and to illuminate dysfunctional HDL, LOOH, MPO serum level changes in patients with acute stroke in the first 24 h in terms of their prognostic superiority to each other and the effect of each on infarct volume and clinical disability. Due to the lack of sufficient studies and projects in this field, our study will be an eye-opener for many researchers in this respect.

## 2. Materials and methods

### 2.1. Study design and patient cohort

This single-center, prospective study was conducted with 100 patients aged 18–80 years who applied to the emergency department with acute ischemic stroke in the first 24 h and were hospitalized in the neurology clinic, and 50 control group patients admitted to the neurology outpatient clinic between April 2020 and September 2020. The study was carried out after obtaining the written consent of each participant. Participants in the control group to be included were selected from individuals without signs of intracranial disease such as stroke, intracerebral hemorrhage, tumor, demyelinating disease, and intracerebral parenchymal or meningeal infection. Persons to be selected for the patient group with a history of other intracranial diseases that could be included in the differential diagnosis of stroke were excluded from the study. To determine the demographic data (i.e. age, sex, race, and chronic diseases) were recorded in the data forms. In the clinical evaluations of the patients, early disability was determined for each patient using the hospitalization/discharge NIHSS score and the discharge mRS.

### 2.2. Imaging analysis

Infarction volume was calculated in the diffusion (DWI) sequence in cranial magnetic resonance imaging (MRI) of all patients. The area calculation of the infarct was made with the Freehand ROI tool in the DWI sections using the AW Volume Share 7 workstation, this area information obtained from each section was collected and then the volume information was created by multiplying the section thickness by 5 mm and the interslice gap of 1 mm (6 mm). A clinical neurologist (E.D.U.) experienced in stroke imaging independently performed an image review. Reviewers were blinded to biological, therapeutic, and imaging follow-up outcomes but had details about the demographic data and clinical appearance of the cases. Neither of the reviewers had taken part in the selection of the patients.

### 2.3. Preparation of blood samples and measurements

Five milliliters of blood samples were taken to the heparinized vacutainer tube from the stroke patients (within the first 24 h of stroke) and the control group patients. The blood samples were immediately centrifuged for 10 min at a speed of 1300 × *g* in a Hettich centrifuge device and the plasmas were separated. Plasma samples were stored in the freezer at −80 °C until the measurement time.

#### 2.3.1. Evaluation of plasma dysfunctional HDL level and PON1 activity

Dysfunctional HDL measurement was based on the principle of detecting the dysfunction of HDL in the rate of MPO/PON by the method defined by Haraguchi et al. [10]. PON1 measurement was carried out based on spectrophotometric measurement of 4-nitrophenol, which is formed as a result of enzymatic hydrolysis of paraoxon used as a substrate, at a wavelength of 412 nm. For basal PON activity measurement, 50 μL of serum 2 mmol/L paraoxon (O, O-diethyl-Op-nitrophenyl phosphate. Sigma chemical, St. lois, Mo., USA), 1-mL Tris-HCL (Tris(hydroxymethyl) aminomethane) was added to the buffer (100 mmol/L, Ph:8). PON enzyme activity was used as a molar extinction coefficient of 17, 1000 M-1 cm. The amount of 1 nm of 4-nitrophenol PON to be formed per minute per milliliter of serum was accepted as a unit.

#### 2.3.2. Evaluation of plasma myeloperoxidase level

MPO levels were measured using commercially available Human MPO ELISA kits (Cayman Chemical, Michigan, USA). For the human MPO ELISA kit, the detection interval in the analysis of samples is 0.2–10 ng/mL and its sensitivity is 0.2 ng/Ml. The within-run and interstudy CV (coefficient of variation) is 3.83% and 8.13%, respectively. In the first step, wells covered with MPO were encompassed with diluted patient samples. In the second stage, the enzyme affected by antihuman IgG was used and incubation was performed again. The intensity of this color, which was formed by providing a color formation as a result of the enzymatic reaction, was scaled in direct proportion to the antibody concentration created against MPO.

#### 2.3.3. Evaluation of plasma lipid hydroperoxides level

LOOH was measured with the xynelol orange method. The method was applied according to the principle of oxidation of Fe+2 (Ferrous sulfate) to Fe+3 (ferric polymaltose) by LOHH in the acidic environment described by Jiang et al. [11]. The Fe+3 formed was measured spectrophotometrically with a purple dye with maximum absorbance at 550–570 nm by forming a complex of xynelol orange (o-cresosulfonephtalein-xynelol 3,30-bismethylimino-diacetic acid; XOF). Roche brand Cobas C 501 automatic analyzer was used for spectrophotometric measurements.

### 2.4. Statistical analysis

SPPS 25 (IBM Corp. Released 2017) statistical package program was used to evaluate the data. In the study, descriptive statistics (mean, standard deviation, median, minimum-maximum values, number and percentile) were given for categorical and continuous variables. The homogeneity of the variances was checked with the Levene test. Normality assumption was checked with the Shapiro–Wilk test. For the differences between the two groups, Student’s t-test was used if the parametric test prerequisites are met; if not, the Mann–Whitney U-test was used. Relationships between categorical variables were analyzed with Fisher’s exact test and Pearson’s chi-squared test. The relationship between two continuous variables was evaluated with the Pearson correlation coefficient and Spearman correlation coefficient. Logistic regression analysis was used when the dependent variable was two-level. Cut-off scores of patients and healthy individuals according to measurement parameters were evaluated by ROC analysis. AUC value, sensitivity, and selectivity values ​​were calculated. A p < 0.05 level was considered statistically significant.

## 3. Results

### 3.1. Baseline data

One hundred patients and 50 healthy volunteers were included in the study. Fifty-seven percent of the patients were female (n: 57) and 48% (n: 48) were male; 52% of the control group was female (n: 26) and 48% male (n: 24). The mean age was 66.31 ± 11.947 in the patient group and 64.9 ± 6.11 in the control group. Sex distribution and mean age were similar between groups (respectively p = 0.561; p = 0.121).

### 3.2. Clinical assessment

In the patient group, admission NIHSS, discharge NIHSS, and discharge mRS scores were evaluated and infarct volume (ROI (cm^3^)) was calculated. NIHSS scores were analyzed in 5 categories; 0–5 were minor, 5–15 were moderate, 15–20 were severe, 20–42 were very severe and those of 42 and above were considered exitus. Discharge mRS scores were examined in 5 groups: 0–1 good, 2–3 moderate, 4–5 were bad, and those of 5 and above were accepted as exitus. The median (IQR) NIHSS score at arrival was 6 (1–23), the median (IQR) NIHSS score at discharge was 6 (0–23), and the mean mRS at discharge was 3 (0–5), 11 of the patients had a stroke history and 7 had a stroke in their family history. Seventy-five percent (n: 75) of the patients had hypertension, 52% (n: 52) were smoking, and 31% (n: 31) had hyperlipidemia. Great artery atherosclerosis was the most common type of stroke.

### 3.3. Biochemical assessment

Serum total cholesterol (TC), HDL, low density lipoprotein (LDL), TG, PON, LOOH, MPO, and MPO/PON values ​​were evaluated and compared in the patient and control groups. Except for TG, other measured parameters differed statistically between the groups. While TC, LDL, LOOH, MPO levels and MPO/PON ratio were lower in the healthy control group; HDL and PON were lower in the patient group. Although the TG level was found to be high in stroke patients, it did not reach statistical significance (p > 0.05) (Table 1).

### 3.4. Biochemical assessment, radiological outcome, and clinical disability correlations

Correlation analyses were used to determine a relationship between serum TC, HDL, LDL, TG, PON, LOOH, MPO, MPO/PON levels and admission/discharge NIHSS, discharge MRS, and infarct volume (ROI (cm^3^)). A weak negative correlation was found between LOOH and discharge mRS (respectively; r = −0.204; r = −0.244, p < 0.05). Infarct volume and TG levels were negatively correlated, MPO/PON ratio was positively correlated (respectively; r = −0.226, r = 0.204, p < 0.05) (Table 2).

Logistic regression analyses performed to reveal the factors that may affect stroke severity (NIHSS score 6 and above) and early disability (mRS score 2 and above) did not show statistical significance for lipid profile, LOOH, PON, MPO, MPO/PON. However, for stroke severity, hypertension (OR = 3.287, 95% CI = 1.080–10.09, p = 0.036), presence of diabetes (OR = 0.292, 95% CI = 0.089–0.962, p = 0.043), and HbA1c levels (OR = 1.487, 95% CI = 1.096–2.019, p = 0.011); for early disability, diabetes history (OR = 0.011, 95% CI = 0.000–0.893, p = 0.044), HbA1c levels (OR = 5.059, 95% CI = 1.222–20.939, p = 0.025), infarct volume (OR = 1.027, 95% CI = 1.008–1.047, p = 0.005), and NIHSSa score (OR = 21,646, 95% CI = 3.113–150.524, p = 0.002) were determined as independent risk factors (Table 3).

ROC curve analyses to show the sensitivity and specificity of serum parameters in predicting stroke severity and early disability revealed statistical significance only for LOOH. When the cut-off point was accepted as 2, LOOH was found with 68.49% sensitivity and 55.56% specificity (p = 0.0375); when the cut-off point is accepted as 4, it can predict disability with 76.00% sensitivity and 57.33% specificity in the mRS score for early disability (p = 0.0377) (Table 4) (Figure).

## 4. Discussion

In this study, we examined the augmentation of lipoprotein peroxidation, serum oxidative stress parameters, and dysfunctional evolution of HDL caused by rheological changes in the cerebrovascular system in the first 24 h of ACI with clinicoradiological and biochemical correlations. An increase in oxidative stress parameters and HDL’s dysfunctionality was demonstrated in the first 24 h which was thought to be an independent risk factor in determining clinical disability in ACI. A positive correlation was found between dysfunctional HDL and infarct volume and correlated with poor radiological outcomes.

Ischemic stroke develops as a result of three basic mechanisms: thrombotic, embolic, and hemodynamic. Atherosclerosis is the most important basic process of the thrombosis and mostly develops because of factors such as hypertension, diabetes, dyslipidemia, and smoking [12]. Among the demographic data of 100 patients diagnosed with acute ischemic stroke, atherosclerotic stroke was found to be the first (87%) of stroke types and the most common risk factors were hypertension (46%), dyslipidemia (46%), and diabetes mellitus (34%).

The atherosclerotic process adversely affects the rheological balance in both macro and microvascular areas in dyslipidemic patients, and even this process begins 2 or more years before the onset of stroke [13,14]. Hoshino et al. [15] investigated the serum lipid profile in 792 patients with ACI and found that the rate of atherogenic dyslipidemia was 12.2% in the first week, the rate of intracranial artery stenosis was higher in these patients, and major adverse cardiovascular events (24.5%) and ischemic stroke (16.8%) were more common at the end of 1 year. In the present study, dyslipidemia was present in 46% (n: 46) of the patient group. In addition, TC and LDL values showed statistically significant differences compared to the control group. Except for serum TG, other findings were higher and serum HDL level was lower in the patient group. There was a weak negative correlation between serum TG levels and infarct volume (r = 0.246, p < 0.05). In this respect, a similar study was conducted by Pikija et al. [16] in 121 ACI patients. The relationship between infarct volume and fasting serum TG level taken within the first 24 h after hospitalization was investigated, and an independent relationship was shown between fasting serum TG levels and lower infarct volume. Our findings complement and align with reports based on clinical outcomes [17,18].

MPO is an acute phase reactant, generating free radicals and diffuse oxidants with antimicrobial activity, and promoting oxidative damage to major tissues, including atherosclerotic lesions at inflammation sites [19,20]. Besides the proinflammatory effect, new evidence points to a role for MPO-generated oxidants as participants in rendering HDL dysfunctional within human atherosclerotic plaque by binding to HDL within human atherosclerotic lesions via specific interactions with apolipoprotein (apo) A-I, the predominant protein of HDL [21]. In the study of Cojocaru et al. [22], in acute stroke patients, MPO levels looked at in the first 24 h were found to be significantly higher and thought to be used for diagnostic purposes. In our study, MPO activity was higher in the patient group (96.28 **±** 66.94) (p < 0.01) which shows that oxidative stress in the early acute phase both sets the stage for ischemia by affecting the rheological order quantitatively and also affects the antiinflammatory mechanisms qualitatively since this parameter is an indirect indicator of dysfunctional HDL formation.

HDL particles, which perform many functions, such as the regulation of cholesterol and protein transport, are considered “functional”, while the otherwise called “dysfunctional”. Low HDL-C levels are associated with the development of cardiovascular diseases and pose a risk for recurrent cerebral ischemic events [23,24]. Characteristic features of dysfunction include decreased activity in its function and components, such as PON1 and apoA-1. In this context, HDL function, PON1 enzyme activity, and eNOS expression values will guide the diagnosis of endothelial dysfunction [25]. In a study by Haraguchi et al., the correlation between MPO and PON and the formation of dysfunctional HDL, and in this context, their effects on coronary risk stratification and function of lipoproteins were investigated in 158 coronary artery patients. The high MPO/PON1 ratio was independently correlated with the ischemic effect on coronary arteries and concluded that the MPO/PON1 ratio is associated with HDL dysfunction, which can be used as an independent biomarker [10]. In our study, PON activity was lower (237.78 **±** 124.06) and the ratio of MPO/PON, which affects and alters the function of HDL, was shown to be higher in the patient group (0 **±** 61 **±** 0.71) (p < 0.01). We concluded that this increase will constitute one of the major etiological factors of ACI by creating a series of rheological changes in the cerebrovascular system, from oxidative modifications of lipoproteins to vascular inflammation.

Paraoxanase is a hydrolase enzyme that is synthesized by the liver and released into the serum, where associated with HDL functionality [26]. Reduced activity of PON1 has been implicated in the development of atherosclerosis [27]. Moreover, during this process, MPO generates the oxidants hypochlorous acid and nitrogen dioxide, which can lead to posttranslational modification of PON1, including tyrosine modifications that inhibit PON1 activity [28]. In Chawhan et al.’s study [29], PON1 activity was measured spectrophotometrically in 50 ischemic stroke patients and 50 healthy controls and was found to be decreased significantly in patients (p < 0.001). Kotur-Stevuljevic et al. investigated oxidative stress status, PON1 status, lipids, and high-sensitivity C-reactive protein (hsCRP) in 185 ACI patients and found decreased HDL-cholesterol level and a remarkable fall in PON1 activity than controls (p < 0.001), along with more prominent inflammation [30]. In our study, PON1 was found to be lower in the patient population (p < 0.01, p < 0.05) which shows the imbalance in prooxidative-antioxidative status. In one study, serum PON level, NIHSS score and ischemic volume were compared in terms of stroke risk factors in 34 ACI patients in the first 72–96 h and a significant decrease in serum PON values of the patient group was determined (p < 0.001) [31]. Xu et al. investigated the relationship between serum PON1 activity and functional outcome in 336 ACI patients and found a significant decrease in the mRS score across serum PON1 quartiles which concluded that serum PON1 activity may be an independent predictor of the functional outcome in ACI [32]. Moreover, Ferretti et al. showed a negative correlation between the PON1 activity and the values of the NIHSS in 49 ACI patients [33]. Our study showed a negative relationship between PON1 activity and arrival NIHSS (r = 0.204 p < 00.05) which confirms that subjects with lower PON1 activity are more exposed to clinical disability.

LOOH is the first by-product of oxidized lipids and serum level determination is important [34]. Ferretti et al. [33] compared paraoxanase activity and plasma lipid hydroperoxide levels in 49 stroke patients and demonstrated that the activity of PON1 was significantly lower (p: 0.001) and the levels of lipid hydroperoxides were significantly higher in stroke patients (p: 0.001). Moreover, a positive correlation between the individual values of lipid hydroperoxides and the NIHSS score was found, which confirms a relationship between oxidative damage and neurological impairment in patients. We measured the LOOH level as an index of lipid peroxidation and was found to be higher in the patient group (8.03 **±** 4.93) than in the control group (4.67 **±** 2.22) (p < 0.01). The ROC analysis revealed that the LOOH parameter was a good predictor of functional outcomes.

Derex et al. showed that arrival NIHSS scores were correlated with acute DWI lesion volumes (r = 0.71), and the NIHSS score was associated with arterial occlusion [35]. In another study, infarct volume and NIHSS scale were monitored over 7 days in 375 ACI patients with known diabetes history or HbA1c value ≥6.5%, and multivariate regression analysis showed that high glucose level was independently associated with infarct volume growth in all patients [36]. In addition, high glucose levels have been proven to be independently associated with neurological worsening in all patients. Logistic regression analyses showed diabetes and elevated HbA1c levels to be an independent predictor of stroke severity (OR = 1.487, 95% CI = 1.096–2.019, p = 0.011). Both HbA1C and infarct volume were found to be effective in prepredicting early disability (respectively OR = 5.059, 95% CI = 1.222–20.939, p = 0.025; OR = 1.027, 95% CI = 1.008–1.047, p = 0.005).

As for the limitations of the study, the present study was based on a detailed interview with the patient (and/or a caregiver) that was carried out within 24 h of hospital admission for acute stroke. The parameters examined within the study do not include the significance level of the parameters that have reached statistical significance in subacute or chronic return of stroke. In addition, the limited patient population included in the study brings to mind the idea that different results can be obtained when similar studies are conducted with larger patient groups of different ethnic origins.

## 6. Conclusion

In summary, ACI process is the result of dysregulation of the oxidative/antioxidative process and MPO plays a role in the oxidation of LDL also a decrease in HDL-related functional PON1, which indicates HDL dysfunction. An increase in MPO enzyme activity leads to an increase in dysfunctional HDL. Although cardiovascular disease publications are remaining valid that lipid-lowering drugs reduces stroke recurrence, there is no proven study yet that reveals whether stroke severity, ischemia, HDL level of atherosclerosis or its functional capacity are related. In this respect, our study will be seminal in terms of both treatment principles and the detection of pathophysiological and therapeutic approaches. Moreover, our study is valuable to show the correlation between dysfunctional HDL, infarction volume and stroke severity in stroke patients. The thought that the qualitative value may be more important than the quantitative measurement of serum lipid levels seems to deserve consideration. Therefore, dysfunctional HDL should be included in the lipid profile test battery when evaluating stroke patients.

## Figures and Tables

**Figure:**
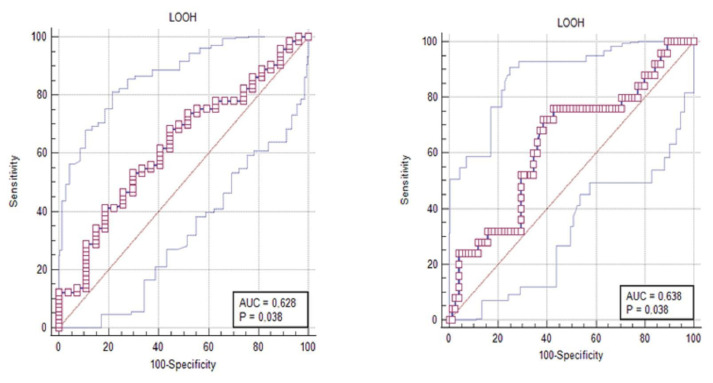
ROC curve analysis of LOOH for mRS ≥ 2 (left) and mRS ≥ 4 (right).

**Table 1:** Lipid, PON, MPO, LOOH, MPO/PON parameters (mg/dL) in the patient and control groups.

	Control group (n = 50) mean ± SD [median (min.–max.)]	Patient (n = 100) mean ± SD [median (min.–max.)]	p-value
TC	152.32 ± 36.51 [163.00 (63.00–214.00)]	176.33 ± 39.55 [170.00 (94.00–300.00)]	0.007 1**
HDL	43.94 ± 19.04 [43.50 (9.00–95.00)]	37.75 ± 11.86 [35.00 (19.00–106.00)]	0.020 1*
LDL	85,32 ± 29,07 [89.00 (15.00–145.00)]	112.52 ± 36.96 [109.00 (21.00–222.00)]	0.001 1**
TG	120.08 ± 53.29 [106.00 (50.00–327.00)]	156.66 ± 101.99 [118.00 (54.00–555.00)]	0.060
PON	295.51 ± 141.77 [271.26 (74.15–558.28)]	237.78 ± 124.06 [209.33 (26.82–471.49)]	0.014 1*
LOOH	4.67 ± 2.22 [4.01 (2.28–16.92)]	8.03 ± 4.93 [6.14 (2.66–24.53)]	0.001 1**
MPO	43.16 ± 32.69 [34.94 (6.52–184.55)]	96.28 ± 66.94 [76.49 (28.79–312.40)]	0.001 1**
MPO/PON	0.19 ± 0.19 [0.13 (0.04–1.05)]	0.61 ± 0.71 [0.35 (0.09–4.71)]	0.001 1**

** p < 0.01

* p < 0.05

1 Mann–Whitney U test

**Table 2:** Correlation between stroke severity, early disability, infarct volume, and lipid profile, PON, LOOH, MPO, MPO/PON.

		Arrival NIHSS	Discharge NIHSS	Discharge mRS	ROI (cm^3^)
TC	R	0.016	0.006	−0.013	−0.196
	P	0.872	0.954	0.896	0.051
HDL	R	−0.029	−0.052	−0.024	−0.090
	P	0.771	0.608	0.816	0.376
LDL	R	0.075	0.045	0.059	−0.114
	P	0.456	0.655	0.563	0.259
TG	R	−0.051	0.026	−0.049	−0.226*
	P	0.614	0.799	0.630	0.024
PON	R	−0.204*	−0.150	−0.093	−0.194
	P	0.042	0.137	0.359	0.054
LOOH	R	−0.195	−0.170	−0.244*	−0.111
	P	0.051	0.090	0.015	0.273
MPO	R	0.011	−0.041	−0.020	0.108
	P	0.912	0.682	0.845	0.285
MPO/PON	R	0.100	0.032	0.008	0.204*
	P	0.324	0.749	0.933	0.042

* p < 0.05

** p < 0.01

1 Spearman’s correlation coefficient

**Table 3:** Logistic regression analysis for possible factors predicting stroke severity and early disability.

	Stroke Severity (NIHSS > 6)			Disability (mRS > 2)		
	Odds ratio	%95 CI	p	Odds ratio	%95 CI	p
HT	3.287	1.080–10.009	0.036 *			
Diabetes mellitus	0.292	0.089–0.962	0.043 *	0.011	0.000–0.893	0.044 *
HbA1c	1.487	1.096–2.019	0.011 *	5.059	1.222–20.939	0.025 *
ROI (cm^3^)				1.027	1.008–1.047	0.005 **
NIHSS				21.646	3.113–150.524	0.002 **
−2 Log likelihood = 120.620 Cox & Snell R square = 0.159 Nagelkerke R square = 0.213 *x^2^* = 17.369				−2 Log likelihood = 21.008 Cox & Snell R square = 0.616 Nagelkerke R square = 0.894 *x^2^* = 95.644		

** p < 0.01

* p < 0.05

**Table 4:** ROC curve analysis of LOOH for mRS ≥ 2 (left) and mRS ≥ 4 (right).

	mRS	Break point	Sensitivity	95% CI	Specificity	95% CI	Area under the ROC curve (AUC)	95% CI	p
LOOH	mRS ≥ 2	7.22	68.49	56.6–78.9	55.56	35.3–74.5	0.628	0.526–0.723	0.0375
LOOH	mRS ≥ 4	6.2	76.00	54.9–90.6	57.33	45.4–68.7	0.638	0.535–0.731	0.0377
